# Developing relevant assessments of community-engaged research partnerships: A community-based participatory approach to evaluating clinical and health research study teams

**DOI:** 10.1017/cts.2023.544

**Published:** 2023-05-11

**Authors:** Elias Samuels, Donald Vereen, Patricia Piechowski, Athena McKay, E. Hill De Loney, Sarah Bailey, Luther Evans, Bettina Campbell, Yvonne Lewis, Ella Greene-Moton, Kent Key, DeWaun Robinson, Arlene Sparks, Ellen Champagne, Susan Woolford

**Affiliations:** 1 University of Michigan, Michigan Institute for Clinical & Health Research, Ann Arbor, MI, USA; 2 Health Awareness Center, Flint, MI, USA; 3 Community Based Organization Partners, Flint, MI, USA; 4 Bridges into the Future, Flint, MI, USA; 5 All Faiths Health Alliance, USA; 6 Anders Associates Flint, MI, USA; 7 Healthy Flint Research Coordinating Center Flint, MI, USA; 8 National Center for African American Health Consciousness, Flint, MI, USA; 9 Michigan State University. College of Human Medicine, East Lansing, MI, USA; 10 Artistic Visions Flint, MI, USA

**Keywords:** Community engagement, evaluation, finance, program evaluation, community-based participatory research

## Abstract

**Background/Objective:**

In 2017, the Michigan Institute for Clinical and Health Research (MICHR) and community partners in Flint, Michigan collaborated to launch a research funding program and evaluate the dynamics of those research partnerships receiving funding. While validated assessments for community-engaged research (CEnR) partnerships were available, the study team found none sufficiently relevant to conducting CEnR in the context of the work. MICHR faculty and staff along with community partners living and working in Flint used a community-based participatory research (CBPR) approach to develop and administer a locally relevant assessment of CEnR partnerships that were active in Flint in 2019 and 2021.

**Methods:**

Surveys were administered each year to over a dozen partnerships funded by MICHR to evaluate how community and academic partners assessed the dynamics and impact of their study teams over time.

**Results:**

The results suggest that partners believed that their partnerships were engaging and highly impactful. Although many substantive differences between community and academic partners’ perceptions over time were identified, the most notable regarded the financial management of the partnerships.

**Conclusion:**

This work contributes to the field of translational science by evaluating how the financial management of community-engaged health research partnerships in a locally relevant context of Flint can be associated with these teams’ scientific productivity and impact with national implications for CEnR. This work presents evaluation methods which can be used by clinical and translational research centers that strive to implement and measure their use of CBPR approaches.

## Introduction

The National Center for Advancing Clinical and Translational Science at the National Institutes of Health funds a consortium of Clinical and Translational Science Award (CTSA) centers located in over 60 universities and research institutions nationwide. A key goal of the CTSA Consortium is to accelerate the process of translating scientific discoveries into improvement in human health through community engagement (CE) [1,2]. The COVID-19 pandemic’s disproportionate impact on the health and healthcare of minority communities across the country deepened the investment in CE being made by research centers in the CTSA Consortium, including associated funding to study the long-term effects of the COVID-19 pandemic [3,4].

This paper presents an evaluation of health research partnerships supported by a CTSA hub, the Michigan Institute for Clinical and Health Research (MICHR). A community-based participatory research (CBPR) approach was used to evaluate community-engaged research (CEnR) partnerships working across the spectrum of CE [5,6]. This study contributes to translational science by demonstrating how CEnR partnerships can be evaluated by health research centers using CBPR approaches. The results of this approach also contribute to a burgeoning line of research demonstrating that CE health research partnerships have national relevance for impact on community health and healthcare [7,8]. We achieve this by focusing on locally relevant approaches to evaluation which can be used to inform the assessment of CEnR on a broader scale. While there is no single way that CEnR partnerships “work,” this paper provides an example of how CTSA-supported CEnR partnerships geographically centered in Flint were evaluated, which may help inform evaluation of other teams. We have attempted to provide sufficient detail to allow clinical and translational scientists seeking to learn from this example to compare their and their partners’ circumstances and goals to those presented here, thus enhancing their ability to draw conclusions about the relevance of this paper to their own research and scientific goals.

Many CTSA hubs have worked to develop comprehensive assessment models to use in the evaluation of CE programs and services [7]. This study builds on the long-term accomplishments of existing health research partnerships in Flint, Michigan. The assessment used for this study was developed by combining and recategorizing existing CEnR partnership evaluation tools using a concept mapping process [9]. Specifically, this study integrates new quantitative measures of financial management with existing measures of CEnR partnerships that have been validated through empirical research.

At the beginning of the CTSA funding in 2007, MICHR began collaborating with community partners in organizations in Flint, Detroit, and Ypsilanti, MI. Those from Flint were mainly members of the Community Based Organization Partners (CBOP), a nonprofit organization representing over 40 multisector and faith-based community organizations. This collaboration included citizen scientists with years of CBPR experience in Flint dating back to the early 1990s with their participation in the W. K. Kellogg Foundation’s Community Based Public Health Initiative [10].

The authors of this study include MICHR faculty, staff, and CBOP board members and other community partners, who formed a workgroup with the aim of guiding the implementation of health research initiatives involving communities in Flint. The authors met bimonthly starting in 2016 and all the community partners were compensated $25/hour for their time for the duration of the project. This group adopted a CBPR approach to develop and enhance MICHR’s support of health research on the long-standing health disparities in Flint, particularly those revealed and exacerbated by the ongoing Flint water crisis.

### Community-Academic Partnerships Evaluated by This Study

The community-academic teams evaluated in this study were selected by virtue of having received funding via a mechanism for CEnR partnerships developed by MICHR in collaboration with CBOP. This mechanism named the Building Capacity for Research and Action (BCRA) was launched in 2017. BCRA awards funded multi- and transdisciplinary scientific teams that proposed to engage community partners throughout the entire research process. All funded projects were required to focus on community-identified health priorities and to use health measures to evaluate the impact of their project. A scientific committee of community and academic reviewers reviewed the proposals and recommended awardees to MICHR. MICHR made final funding decisions based on the review committee’s recommendations.

The funded teams, which required a community partner from Flint, had a wide range of prior experience, with some building on decades of shared work while others included new collaborators working together for the first time. An innovative element of this mechanism is that non-U-M academics were encouraged to apply for funding. However, most applications came from teams where the academic partner was from the U-M.

The awards were distributed in three rounds during the study period. A total of 16 BCRA project proposals were accepted for funding between 2018 and 2022. Each funded team attended orientation meetings with MICHR specialists in research regulation, administration, finance, CE and program evaluation.

The BCRA awards ranged from $5K to $10K and were all offered for a 12-month project period, with one possible 6-month no-cost extension, in total MICHR awarded $150,996 in BCRA funding. Overall, the funded projects encumbered 34% of their budgets for community partner effort. And among the 12 projects which have completed their work as of August 2022, a total of $62,663 was actually allocated towards community partner expenses, a sum representing 54% of the total proposed budget and 56% of the total amount spent.

Two additional awards for health research projects in Flint are also included in this study. One participating partnership was conducting research on the use of Reiki for the treatment of substance abuse. This project was supported with $5000 in MICHR pilot grant funding, 87% of which was encumbered for community partners and 89% of the total budget spent ultimately went to community partners. The other partnership included in this study received $25,000 in MICHR discretionary funding to engage community members in discussions about the Flint water crisis and their trust in health research.

## Methods

This study is an exploratory evaluation of CEnR partnerships using existing and new survey measures that were categorized into domains through a collaborative concept mapping process [9,12]. Following best practice [13], this evaluation was reviewed and approved by the CBOP’s Community Ethics Review Board (CERB) in Flint, which provided a letter of endorsement for the project. This letter of endorsement was included in the proposal which was reviewed and exempted from oversight by the University of Michigan Medical School’s Institutional Review Board (HUM00156451).

### Developing an Assessment of Partnership Dynamics

The assessment administered to BCRA-funded partnerships was developed using existing assessments (e.g., Wallerstein, 2011 and Israel, 2008) as well as novel questions about financial management which were created by the authors. First, validated assessments of CEnR partnerships published in peer-reviewed journals were collected and reviewed for relevance. Over 120 distinct questions derived from published assessments of CEnR were identified [1,5,6,14–29].

The concept mapping process used for this study involved six steps [9]. These included 1) preparing all existing measures for review, detailing their measurement scale, citing the author, and associating each with the domain utilized by the author, 2) developing new questions to address any resultant gaps among the questions, 3) rating the relevance and appropriateness of all questions for use in this study, 4) grouping all of the questions by cross-cutting key domains of community-engagement developed by the study team, 5) analyzing the relationships between the questions, domains, and the teams’ ratings, and 6) developing a comprehensive assessment for use in pilot testing.

The use of established conceptual domains of CEnR partnerships enabled the study team to categorize and compare the existing assessment measures. Novel questions about financial management were also developed to compliment the few questions grouped into one domain. The mapping process resulted in a comprehensive set of survey questions grouped into six broad domains of community-engaged partnerships, including 1) Partnership Background & Sustainability, (2) Communication, (3) CE, (4) Decision Making & Trust, (5) Team Finances, and (6) Impact.

The Partnership Background and Sustainability Domain includes existing measures of the composition, diversity, and structure of health research partnerships, including the community-identified health priorities of focus and the degree of CE on the spectrum of CEnR [5,6]. This domain also includes questions regarding partners’ support for the partnership over time and the sustainability of the partnership [14]. The CE Domain contains questions including ones about the shared credit for, and ownership of research achievements [15], and assessments of the Principles of CE [14–16].

The Communications Domain includes measures derived from several existing assessments. These include measures of how community and academic partners interacted during meetings, conversed in respectful and productive ways, and agreed with their teams’ mission, priorities, and work strategies [14,15,17,18]. The questions regarding Decision-Making and Trust Domain include questions regarding partners’ support of the decisions made by their team, their inclusion in those decisions, their comfort with the process, and their feeling pressured to go along with decisions of the group [14,19]. A measure of trust is also included which characterizes the type of trust that existed within their partnership using an ordinal scale [2,20].

The Team Finances Domain includes existing questions regarding the felt needs of community partners, their access to the benefits of health research partnerships, and the equity with which those benefits were distributed [14,15,19]. Novel questions about the allocation of financial resources were also developed by the study team. The Impact Domain includes questions regarding the effect of the partnership on the health of local communities, the quality of health care provided to them and the degree to which the partnership advanced community-identified health priorities. These measures also include questions measuring how the partnership benefited the partners overall, and individually, including through subsequent awards and recognition [14,15,19].

Each measure used in the concept mapping process was reviewed in two focus group sessions of CEnR partnerships using a semi-structured protocol. Participants completed the assessment in advance and then discussed the relevance of each question to their past research experience. The discussions were recorded, and the transcripts were analyzed to identify participants’ recommends for changes to the survey. As shown in Table 1, after making these revisions, the resultant survey questionnaire was reduced from 137 questions down to 67.

**Table 1.** The number of survey question by domain and study phase

*Survey Form Categories*	*Concept Mapping Process*	*Focus Groups*	*Resultant Survey*
Partnership background and sustainability	8	8	8
Communication	28	20	15
Principles of engagement	17	12	11
Decision-making and Trust	42	29	7
Team finances	14	13	10
Impact	28	19	16
Total number of questions	137	101	67

The resultant survey was sent to every member of each participating study team in 2019 and in 2021. Participants received survey invitations and forms which were personalized with their names as well as the name of the partnership which they were being asked to evaluate. Following the initial survey invitation two reminders were sent 1 week apart to all nonresponders.

Two 2-tailed t-tests (assuming unequal variances) were conducted to identify statistically significant differences, both between responses in 2019 and in 2021, but also between community and academic partners responding within each year. ANOVAs were conducted to look for differences in categorical measures. More rigorous longitudinal analyses of differences among people over time could not be conducted due to the small number of academic and community partners who responded to both iterations of the survey.

For formative evaluation, the differences in the perceptions of community and academic partners were quantified; each team received their data, and anonymized reports of the comparative results were provided to all participants in 2021. Further feedback about the financial management practices of the funded CEnR partnerships was subsequently collected through an anonymous online survey using open-ended questions. Invitations to complete the survey were sent to members of all funded teams in 2022.

## Results

In 2019, a total of 14 funded study teams received invitations, and at least one member of each team responded. Eight teams (57%) returned responses from at least one academic partner and at least one community partner. A total of 56 individuals received survey invitations in 2019 of which 30 responded, representing a 54% response rate. In 2021, a total of 16 funded study teams received survey invitations, to which at least one member of all but two teams responded, and nine teams (56%) returned responses from at least one academic partner and at least one community partner. A total of 62 individuals received survey invitations in 2021 of which 31 responded, representing a 50% response rate.

Nineteen individuals responded to both surveys, collectively representing 13 of the study teams participating. However, the small number of consistent respondents across years prevents robust longitudinal analyses of statistical difference from being conducted at this time. Eleven individuals (about 18%) responded to the anonymous survey about financial management that was administered in 2022. These survey response rates range from sufficient to high, as specified by Daikeler and colleagues (2020), particularly considering the mitigating factors associated with distributing email invitations to online surveys [30].

These respondents’ survey data were analyzed to identify statistically significant differences between academic and community partners in 2019 and 2021 and differences between respondents by year. Substantive differences were found in respondents’ perceptions of their teams’ 1) Background & Sustainability, 2) CE, 3) Communication, 4) Decision-making & Trust, 5) Team Finances, and 6) Impact. The following sections and tables present each of these sets of results in turn.

Notably, out of the 195 t-tests performed for this study comparing community partner perception to academic partner perceptions of the functioning of their partnership, only six statistically significant differences were found. Five of these statistical differences were related to Team Finances or the Sustainability of the partnership. One statistically significant difference addressed a question assessing the quality of communication within partnerships.

### Partnership Background

When asked to describe their partnership from a list of options, 77% selected to the 2019 survey described their collaboration as being a CEnR partnership in which, “the community provides input, or consults, about critical aspects of the research process, such as research questions, project design, project objectives, data analysis, dissemination, translation of findings” with the remainder (23%) indicating their research was also “placed somewhere in the local vicinity of the community where people are engaging within the context of the physical spaces of the community receiving the service” [5]. The partnerships represented by the respondents’ focused on clinical and health issues including aging populations, hunger, urban populations, men’s health, women’s health, mental health, poverty, youth and adolescent populations, social isolation, sexual and gender minorities, racism, and substance abuse.

A little under half (45%) of the survey respondents in 2021 were paid members of their partnerships, and a greater proportion of respondents (54%) to the 2019 survey were paid members. Half of the academic partners responding in 2019 reported that they had been in their partnership for more than 2 years, compared with only 19% of community partners, who largely reported being in their partnership for less than 2 years. Only 15% of the respondents to the 2019 survey indicated their partnership had been initiated by an academic partner with the rest reporting their partnerships were initiated by the community partner (27%), or jointly by community and academic partners (58%).

As shown in Table 2, although partners indicated that they were committed to sustaining their partnerships with no or low funding, their responses to the statement became less positive between 2019 and 2021 (*µ* = 4.2 in 2019, *N* = 17; *µ* = 6.3 in 2021, *N* = 19). They also remained satisfied with their team’s attention to financial sustainability and evaluation of mutually beneficial funding opportunities. Although there was a statistically significant difference found between the academic and community partners responding to this question in 2021, their rates of commitment remained high on both iterations of the survey (see Table 2). A statistically significant difference was found between the respondents to the 2019 and 2021 surveys. All respondents in 2019 survey expressed a stronger commitment to sustaining their partnerships after their funding ended compared to respondents in 2021 (*µ* = 4.3 in 2019, *N* = 26; 2021 *µ* = 3.6 in 2021, *N* = 31, *t* (48) = −3.2, *p* = .003).

**Table 2.** Partnership sustainability*

	2019	2021
	*N* = 26	*N* = 31
I am committed to sustaining the community/academic relationship with no or low funding. [14]	4.4	3.7
This partnership is likely to continue forward after this funding is over. [14]	4.4	3.6
Our partnership carefully evaluates funding opportunities to make sure they meet both community and academic partners' needs. [14]	4.1	3.8
*1 = Strongly disagree, 2 = Disagree, 3 = Neither disagree nor agree, 4 = Agree, 5 = Strongly agree*		

*Minor changes to the wording of some cited questions were made, as described in the methods section concerning the concept mapping process.

### Principles of Engagement

Respondents were asked to indicate the extent to which their teams used eight well-established principles of CE [1]. Although the partners reported that many of the principles were not used as actively in 2021 compared to 2019, their responses indicated that the teams mostly follow each of the principles (Table 3). The measures showing the greatest changes in magnitude related to partners’ increasing belief that their partnership shares knowledge and findings to all members and involves them all equitably in the dissemination process, which rose from an average 4.25 in 2019 (*N* = 26) to 4.35 in 2021 (*N* = 29). In addition, in both years participants reported their partnership consistently facilitates equity in all phases of its research, balances research and social benefits, and fits local community cultures and norms.

**Table 3.** Principles of engagement*

	2019	2021
	*N* = 26	*N* = 29
This partnership builds on resources and strengths in the community. [1–2,14,16]	4.6	4.5
This partnership facilitates equitable partnerships in all phases of the research. [1–2,14,16]	4.3	4.3
This partnership helps all partners involved to grow and learn from one another. [1–2,14,16]	4.5	4.3
This partnership balances research and social action for the mutual benefit of all partners. [1–2,14,16]	4.3	4.3
This partnership emphasizes what is important to the community (environmental and social factors) that affect well-being. [1–2,14,16]	4.6	4.5
This partnership disseminates knowledge and findings to all partners and involves all partners in the dissemination process. [1–2,14,16]	4.3	4.4
This partnership views community-engaged research as a long-term process and a long-term commitment. [1–2,14,16]	4.7	4.6
This partnership fits local/cultural beliefs, norms, and practices. [1–2,14,16]	4.5	4.5
*1 = Not at all, 2 = A little, 3 = Sometimes, 4 = Mostly, 5 = To a great extent*		

*Minor changes to the wording of some cited questions were made, as described in the methods section concerning the concept mapping process.

Respondents in both years reported that their partnership consistently practiced co-learning, although this question returned the greatest decline of all eight principles of CE measured between 2019 and 2021. The participants further indicated that their partnerships build on community resources and strengths. They also view CEnR as a long-term commitment. However, both measures also declined over the 2 years.

Importantly, in both years, the respondents’ confirmed that their partnerships typically followed all eight principles of CE measured in this study. These consistently positive perceptions of CE are to be expected with the respondents representing a spectrum of CEnR partnerships. However, the trends among these measures of engagement are mixed (Table 3).

### Communication

In contrast to their perceptions of CE, respondents reported holding less positive perceptions of their team’s communication on all but one of the associated measures in the survey. Although the opinions held by the responding partners did not become negative throughout, their ratings of their partnership’s communication declined on almost every measure from 2019 to 2021 (Table 4). The greatest declines were reported in teams’ ability to work together to resolve disagreements and in their ability to reach consensus on the strategies used to pursue priorities. Respondents’ positive opinions about the occurrence of constructive arguments on their team slightly declined during this time as well, particularly regarding the occurrence of disrespectful remarks made during team meetings.

**Table 4.** Communication*

	2019	2021
	*N* = 23	*N* = 27
We showed positive attitudes towards one another. [14]	4.6	4.5
Everyone in our partnership participated in our meetings. [14]	4.3	4.1
We listened to each other. [14]	4.5	4.5
Arguments that occurred during our meetings were constructive. [14]	3.9	3.9
When disagreements occurred, we worked together to resolve them. [14]	4.2	3.9
Even though we did not have total agreement, we did reach a kind of consensus that we all accepted. [14]	4.2	3.9
There were disrespectful remarks made during the meetings. [14]	1.7	2.1
There was hidden or open conflict and hostility among the members. [14]	1.6	2.0
The way the other members said some of their remarks was inappropriate. [14,17]	1.8	2.0
Partnership members communicate effectively with each other outside of meetings. [15]	4.3	3.9
Team members communicate effectively with each other during meetings. [15]	4.5	4.2
Members of our partnership have a clear and shared understanding of the problems we are trying to address. [14]	4.4	4.2
There is general agreement with respect to the mission of the partnership. [14]	4.5	4.3
There is general agreement with respect to the priorities of the partnership. [14]	4.5	4.2
Members agree on the strategies the partnership should use in pursuing its priorities. [14,18]	4.6	4.1
*1 = Strongly disagree, 2 = Disagree, 3 = Neither disagree nor agree, 4 = Agree, 5 = Strongly agree*		

*Minor changes to the wording of some cited questions were made, as described in the methods section concerning the concept mapping process.

Importantly, a statistically significant difference was found between the respondents in 2019 and 2021 regarding their agreement on the strategies the partnership should use in pursuing priorities. In 2021, the respondents were less likely to indicate that there was agreement among their team about the strategies that should be used, compared to 2019 (*µ* = 4.6 in 2019, *N* = 25; *µ* = 4.1 in 2021, *N* = 28, *t* (48) = −2.1, *p* = .040).

### Decision-Making and Trust

The partners’ perceptions of the decision-making process in their partnership remained consistently positive or became slightly less positive over time (Table 5). In both 2019 and 2021, respondents reported often feeling comfortable with the way decisions were being made and supportive of the decisions themselves. Throughout this time, they rarely if ever reported feeling pressured to go along with decisions with which they did not agree or being left out of decision-making. However, they did feel they had been left out of decisions more so in 2021 than they reported in 2019.

**Table 5.** Decision-making*

	2019	2021
	*N* = 25	*N* = 30
Feel comfortable with the way decisions are made in this partnership. [14,19]	4.5	4.5
Support the decisions made by the partnership team members. [14,19]	4.6	4.6
Feel that you have been left out of the decision-making process. [14,19]	1.6	1.7
Feel pressured to go along with decisions of the partnership team even though you might not agree. [14–15,19]	1.5	1.5
*1 = Never, 2 = Rarely, 3 = Sometimes, 4 = Often, 5 = Always*		

*Minor changes to the wording of some cited questions were made, as described in the methods section concerning the concept mapping process.

In both 2019 and in 2021, the respondents rated the level of trust that they had at the start of the partnership, at the current moment, and the level of trust that they anticipated achieving in the future. For each of these questions, the scale ranged from 1 to 7, with definitions of each corresponding level of trust provided. In both years, they tended to report having a consistent level of trust in their team (*µ* = 6.4 in 2019, *N* = 24; *µ* = 6.3 in 2021, *N* = 29). The type of trust respondents had through this period was defined as “proxy trust,” a state in which all members of this partnership are trusted, even if only by proxy due to another team member being viewed as trustworthy.

Respondents’ assessment of the level of trust they had at the beginning of their partnership increased over time (*µ* = 4.3 in 2019, *N* = 24; *µ* = 5.0 in 2021, *N* = 29) while the level of trust they anticipated achieving in the future somewhat declined (*µ* = 6.8 in 2019, *N* = 24; *µ* = 6.5 in 2021, *N* = 29). Most importantly, respondents consistently anticipated that their partnership would reach new levels of “critical reflective trust, in which mistakes and other issues resulting from differences, including differences in culture and power, could be talked about, and resolved in good ways.

### Team Finances

Respondents felt less positively about their study teams’ use of financial resources in 2021 than in 2019. But as shown in Table 6, they consistently reported their partnership was making fair decisions about how its resources were used, and that these resources were distributed in a fair and equitable manner. While they also agreed that they had adequate knowledge of their research budget during this period, they wanted to have more input into their teams’ allocation of resources in 2021 than in 2019. However, these respondents also reported their teams made good use of key resources and time during this period. While their perceptions of how well the partnership used its financial resources and time both declined slightly during this period, their opinion of their teams’ use of in-kind resources improved.

**Table 6.** Team finances*

	2019	2021
	*N* = 26	*N* = 30
Decisions about research resources are made in a fair manner. [15]	4.5	3.7
I have adequate knowledge of research budgets, research resources and how resources are allocated. [15]	4.5	4.0
Thus far, the research team leaders have distributed available resources in a fair and equitable manner. [15]	4.5	4.0
I would like to have more input regarding the allocation of resources. [15]	2.8	3.1
*1 = Strongly disagree, 2= Disagree, 3 = Neither disagree nor agree, 4 = Agree, 5 = Strongly agree*		

	2019	2021
	*N* = 26	*N* = 29
How well your partnership uses financial resources. [14,19]	4.2	4.1
How well your partnership uses In-kind resources. [14,19]	4.2	4.3
How well your partnership uses Time. [14,19]	4.1	3.9
*1 = Makes poor use, 2= Makes fair use, 3 = Makes average use, 4 = Makes good use, 5 = makes excellent use*		

	2019	2021
	*N* = 24	*N* = 28
To what extent were you given an opportunity to be involved in writing the budget for your research project(s)?	8.5	7.6
*0 = no opportunity … 10 = lots of opportunity*		

	*N* = 25	*N* = 30
How equitably was the budget distributed between academic and community partners?	8.3	8.1
*0 = not equitably distributed … 10 = very equitably distributed*		

	*N* = 26	*N* = 31
To what extent were you provided with all the resources you needed to accomplish your work for this project?	8.5	8.2
*0 = none of the resources needed were provided … 10 = All of the resources I needed were provided*		

*Minor changes to the wording of some cited questions were made, as described in the methods section concerning the concept mapping process.

All survey participants were also asked to rate three aspects of their engagement with study team financial management on a sliding 10-point scale. These survey questions were created by the authors as no such validated measure was found to be available. Respondents reported having less opportunity to be involved in writing their team’s research budgets in 2021 compared to 2019 (*µ* = 8.5 in 2019, *N* = 24; *µ* = 7.6 in 2021, *N* = 28. 0 = “no opportunity,” 10 = “lots of opportunity”). They reported that the team’s budget was less equitably distributed in 2021 than they had reported it was in 2019 (*µ* = 8.3 in 2019, *N* = 25; *µ* = 8.1 in 2021, *N* = 30. 0 = “not equitable,” 10 = “very equitable distributions”). Finally, the respondents reported that they had been provided with all the resources they needed to accomplish their work on the study to a lesser extent in 2021 compared to 2019 (*µ* = 8.5 in 2019, *N* = 26; *µ* = 8.2 in 2021, *N* = 31. 0 = “no resources,” 10 = “all of the resources needed”).

Statistically significant differences were found in respondents’ opinions of their team’s finances in 2019 compared to 2021 and between community partners and academic partners’ responses in 2021. Both community and academic partners were less likely to report that their financial resources had been distributed fairly and equitably in 2021 than they had been in 2019 (*µ* = 4.5 in 2019, *N* = 25; *µ* = 4.1 in 2021, *N* = 28, *t* (48) = −2.1, *p* = .040). By 2021, community partners were less likely than academic partners to agree that their teams’ distribution of resources was fair and equitable (Community partners *µ* = 3.6, *N* = 15; Academic partners *µ* = 4.3, *N* = 15, *t* (25) = 2.1, *p* = .035) or to report having adequate knowledge of their research budget (Community partners *µ* = 3.5, *N* = 15; Academic partners *µ* = 4.5, *N* = 15, *t* (20) = 2.6, *p* = .017).

### Impact

The respondents consistently confirmed that their partnerships were impactful on a range of measures used to assess the contributions made by the partnership to community health as well as the work of all the partners involved (Table 7). They consistently indicated that their partnerships impacted the overall health of the community and its overall environment. They similarly reported their partnerships resulted in sustained collaborations between agencies and that it improved the access, delivery, and quality of health services in the community. However, they reported their partnerships having a lower impact on resultant policy change and on their acquisition of additional financial support or receipt of recognition from policymakers and officials.

**Table 7.** Impact*

	2019	2021
	*N* = 23	*N* = 29
Improved the access, delivery, and quality of health services (broadly defined) in the community. [14]	3.2	3.1
Resulted in sustained partnerships among agencies. [19]	3.6	3.6
Resulted in policy changes. [14,19]	2.4	2.3
Improved the overall health status of individuals in the community. [19]	2.9	2.7
Received public recognition or acknowledgment from local policy makers and/or government officials. [19]	2.3	2.1
Resulted in acquisition of additional financial support. [14]	2.9	2.5
Improved the overall environment in the community. [14]	3.0	2.7

	*N* = 23	*N* = 27
The partners' reputation has been enhanced. [14,19]	3.2	3.3
The partners' ability to affect public policy has been enhanced. [14–15,19]	2.6	2.7
The partners' expertise or services have been increasingly utilized. [14,19]	3.0	3.3
The partners' ability to acquire additional financial support has been increased. [14,19]	3.0	2.8
My reputation has been enhanced. [14,19]	2.7	3.0
My ability to affect public policy has been enhanced. [14–15,19]	2.4	2.5
My expertise or services have been increasingly utilized. [14,19]	3.3	2.9
My ability to acquire additional financial support has been increased. [14,19]	2.7	2.7
*1 = Not at all, 2 = To a small extent, 3 = To a moderate extent, 4 = To a great extent, 5 = To a very great extent*		

*Minor changes to the wording of some cited questions were made, as described in the methods section concerning the concept mapping process.

These respondents also increasingly reported that their partnerships were beneficial to the work and abilities of their team. They indicated their partnerships had enhanced their and their partners’ reputations and that the expertise of their partners had been increasingly utilized between 2019 and 2021. However, these respondents also reported that the utilization of their personal expertise declined during this period. Although they reported their partnerships have a small to moderate impact on their personal ability to acquire additional financial support for their research, the respondents felt that their partners had a greater ability to acquire further financial support in 2019 than in 2021.

### Partnership Financial Management Practices

The results of the 2019 and 2021 surveys were aggregated by the authors and sent to each member of the funded partnerships for the purposes of formative evaluation. Each member of the funded teams was then invited to provide anonymous feedback about the financial management practices of their partnership using open-ended questions. The anonymous survey returned dozens of recommendations for financial management practices submitted by community and academic partners.

When asked to share effective approaches to financial management used by their own teams, eight individuals emphasized the value of transparency in discussions about financial resources. One individual noted that their team members had sought, “100% transparency with [the] budget and other resource allocation,” and another reported that their team, “maintained transparent communication through all phases of the project.” A few respondents also emphasized the importance of consistently allocating fair compensation for community partners, including for the time taken to participate in team meetings.

Other best practices in financial management recommended by the respondents included the direct involvement of community members in all financial planning meetings and processes. Considered overall, these recommendations ranged from ensuring that community members are present in discussions about resource allocation to the use of participatory budgeting and “community led” budget formation. These results were used by the authors to identify potential best practices for CEnR teams.

## Discussion

The results of this study demonstrate how the use of a CBPR approach enabled the evaluation of CEnR partnerships supported by MICHR. New and existing measures regarding the management of study team finances were used to evaluate these partnerships and significant differences were found in the participants’ response to some key questions. The funded teams used the results for the purpose of formative evaluation and provided practical, evidence-based recommendations about the financial management of CEnR partnerships in response. These results also demonstrate how partnerships participating in this study believed they were making a positive impact on community health and health care.

The emphasis that this paper places on Flint-focused partnerships is not a limitation of this work. While the CEnR partnerships and survey questions used for this study are clearly locally relevant, the approach and findings of this study have broader implications for the field of CEnR, especially in the context of CTSAs. This work makes two contributions to the field of translational science. First, the results demonstrate the feasibility of using a CBPR approach to evaluate the implementation and impact of health research funding mechanisms for CEnR partnerships [2]. The collaborative process used to develop, pilot, and apply the assessment results is also a best practice which can be used by CTSA hubs to evaluate CEnR partnerships using CBPR methods [1].

Second, the results of this study suggest that the use of more participatory budgeting techniques[31] merits study as a potential best practice for CEnR partnerships. Further research is needed to validate the measures of the Team Finances domain used in this study before the measures of the domain can be used to assess the dynamics of CEnR partnerships. Future studies could validate these measures by testing key hypotheses about their predictive validity, including the following.

H1: Partners’ positive valuations of Team Finances affect the sustainability of their partnerships.

H2: Partners’ positive valuations of Team Finances affect the cultivation of trust within their partnerships.

H3: Partners’ positive valuations of Team Finances contributes to partners’ capacity to conduct clinical and health research.

H4: Partners’ qualitative experiences of the financial management practices used by CEnR study teams will subsequently be manifested in valuations of quantitative measures of Team Finances.

## Limitations

There are four primary limitations of this work. First, because some of the questions about Team Finances were novel, there is no existing record of their validity. Second, the small sample size of this study precludes opportunities for longitudinal analyses required in order to understand why individuals’ perceptions changed over time. Third, no qualitative or mixed-methods evaluation was conducted which could have revealed specific facilitators or challenges to the financial management of these partnerships. The quantitative approach taken in this work focuses narrowly on measures of financial management for CEnR partnerships which can be validated but cannot easily illuminate the specific situations these partnerships encountered in the conduct of their work. Finally, this study was implemented during the midst of nationwide social justice protests following the death of George Floyd, the global rise of the COVID-19 pandemic, and an ongoing water crisis, all of which profoundly affected the work of the Flint-focused BCRA-funded partnerships. Secondary limitations of this study include low-response rates and the possibility that participants may have misinterpreted the survey directions by responding on behalf of their team members instead of themselves as an individual.

## Conclusion

CTSA hubs can accelerate the process of translating scientific discoveries into improvement in health through CE [1,2]. These hubs can have a clear impact on the health and healthcare of communities by supporting studies across the full spectrum of community-engaged clinical and health research [3,4]. And the evaluation of these partnerships is essential to the advancement of translational science [7,8].

The authors chose to use a CBPR approach because it enables the exploration of the dynamics of partnerships that are most relevant to the communities participating in the research [32,33]. The long-standing need for empirical research about work of community-engaged health research partnerships is well-recognized [1,2]. Clinical and translational scientists should build on research on financial management practices of CEnR partnerships [1,31,34–37]. CTSA hubs interested in utilizing a CBPR approach to evaluating CEnR partnerships must carefully consider how their local community and institutional contexts compare to those presented here before using CBPR approaches in similar ways for the partnerships they fund and support.

Studies show that the use of participatory budgeting practices can affect the allocation of public finances, and further that the use of participatory budgeting in health programs can impact measures of community and patient well-being [3–40]. More research is needed to better understand the relationships linking the financial management of CEnR partnerships and their impact on community health. This need may be particularly felt in communities like Flint, Michigan, but it may also be relevant to other communities that are facing ongoing and compounding public health crises [41].
